# Validity of distal radius fracture diagnoses in the Swedish National Patient Register

**DOI:** 10.1186/s40001-023-01314-0

**Published:** 2023-09-09

**Authors:** Hanna Südow, Linda Sjödin, Cecilia Mellstrand Navarro

**Affiliations:** 1 Department of Clinical Science and Education, Södersjukhuset, Karolinska Institute, Stockholm, Sweden; 2 Department of Orthopaedic Surgery, Södersjukhuset, Stockholm, Sweden; 3 Emergency department, Danderyd hospital, Stockholm, Sweden; 4 Department of Hand Surgery, Södersjukhuset, Stockholm, Sweden

**Keywords:** Distal radius fracture, Swedish National Patient Register, Outpatient Register, Inpatient Register, Validation, Epidemiology

## Abstract

Distal radius fractures are one of the most common fractures in adults. More research is needed to establish evidence-based clinical practice guidelines to generate cost-effective and fair fracture treatment. The Swedish National Patient Register is a principal source for population-based epidemiologic studies in Sweden. The validity of some–but not all–diagnoses in the register is high. Little is known regarding the validity of registration of distal radius fractures.

A dataset of cases registered with diagnosis of distal radius fracture (S52.5) or distal radius and ulna fracture (S52.6) were collected from the Swedish National Inpatient and Outpatient Registers. Six cohorts, each containing 240 simple random samples, were constructed. Radiographic reports and medical records were reviewed to confirm or reject the diagnosis as well as, in relevant cases, the surgical intervention. Positive predictive values (PPV) were calculated.

The PPV for distal radius fracture in the register ranges between 92 and 100%, lower if coded as S52.6 and higher if there was an adherent code of surgical intervention. Codes for surgical intervention reached a PPV of 95–100%.

In conclusion, the validity of the codes for diagnosis and the surgical treatment of distal radius fracture is high in the Swedish National Patient Register. According to our results, the register may be used as a reliable data source for population-based research of distal radius fractures.

**Supplementary Information:**

The online version contains supplementary material available at 10.1186/s40001-023-01314-0.

## Background

Distal radius fracture (DRF) is one of the most common fractures in adults [1]. The estimated incidence ranges between 150–289/100 000 person-years globally [1–5], with the incidence rate being higher in the Nordic countries than in other European regions [6]. In the long term in most patients treated adequately for a DRF regain function [7, 8].

In Sweden all patient data is traceable through a personal identification number which is a 12-digit number unique to every Swedish resident. The number is assigned to every resident at birth and to immigrants intending to stay for more than one year [9]. Every health care visit is linked to the patient’s personal number which enables linkage across different medical registers and tracing of patient charts. This is one of the reasons why medical registers in Sweden are exceptionally good for research [9].

The Swedish National Patient Register (SNPR) contains information about inpatient care and specialized outpatient and emergency department visits from both public and private caregivers in Sweden [10]. Administered by the Swedish National Board of Health and Welfare, it consists of an Outpatient Register and an Inpatient Register. It is the largest health register in Sweden and provides a unique opportunity to study diseases in the population due to its nation-wide coverage. Reporting to the SNPR has been mandatory for inpatient and outpatient care since 1987 and 2001, respectively [11]. The register is the principal database for statistics, evaluation, and planning of health care in Sweden and is also a frequently used resource for clinical research. The information collected includes patient data, geographical data, administrative data, and medical data (Table 1). The medical data includes a main diagnosis and secondary diagnoses coded according to the Swedish version of the International Classification of Disease (ICD-10 SE) system [12] and is determined by the treating physician. The data also contain information about intervention coded according to the Nordic Medico-Statistical Committee Classification of surgical procedures (NCSP) which differentiates between surgical methods [13].

**Table 1:** Description of the cohorts in a validation study of the Swedish National Patient Register with regards to ICD-10 code registrations of distal radius fractures

Outpatient register	
Cohort 1	S52.5–Distal radius fracture
Cohort 2	S52.6–Distal radius and ulnar fracture
Cohort 3	S52.5 or S52.6 and NCJ/NDJ29-99 codes for surgical treatment
Inpatient register	
Cohort 4	S52.5–Distal radius fracture
Cohort 5	S52.6–Distal radius and ulnar fracture
Cohort 6	S52.5 or S52.6 and NCJ/NDJ29-99 codes for surgical treatment

To produce reliable epidemiologic research, the data collected in the register must be accurate. The validity of the SNPR has previously been assessed regarding the inpatient component of the register for a broad spectrum of diseases. Analyses have shown an overall high validity with estimated positive predictive values (PPV) of 85–95% [11]. However, most patients with a DRF are treated on an outpatient basis [14] and the outpatient component of the SNPR has only been assessed for a limited number of medical conditions with varying results, with a PPV ranging from 59% [15, 16] to 92% [17–19].

In a recent study, Swärd et al. [16] examined the validity of scaphoid fracture coding in the Inpatient and Outpatient Register. The study showed a low validity of the scaphoid fracture diagnosis in the SNPR with a PPV of 59%, meaning, a high proportion of the patients recorded as having a scaphoid fracture had a false positive diagnosis. It is questionable whether all traumatic orthopaedic diagnoses are valid and reliable in the SPNR.

### Aim

This study aimed to assess the validity of the SNPR by estimating the positive predictive value of the reported ICD-10 code for distal radius fracture and distal radius and ulnar fracture as verified by radiographic examination. As a secondary outcome we aimed to assess the validity in SNPR for NCSP codes of surgical treatment of DRF.

## Methods

### Study population

This is a nation-wide cohort study with six study populations with randomly selected samples each of 240 individuals aged ≥ 18 years. The study period was ten years between January 1st, 2006 and December 31st, 2015. Three cohorts were selected from the Outpatient Register and three from the Inpatient Register (Table 1). In each register one cohort was selected among individuals with a recorded ICD-code S52.5 (distal radius fracture), one among patients with S25.6 (distal radius and ulna fracture) and one with either S52.5 or S52.6 and an addition of a NCSP code of NCJ29-99 or NDJ29-99. Codes analysed in this study were codes for fracture surgery in the hand or forearm: external fixation NCJ/NDJ29, bioimplant NCJ/NDJ39, pinning NCJ/NDJ49, intramedullary implant NCJ/NDJ59, plates and screws NCJ/NDJ69, screws only NCJ/NDJ79. Combinations of methods and very rare surgical methods are coded as undefined or combined method NCJ/NDJ89-99.

**Fig. 1:**
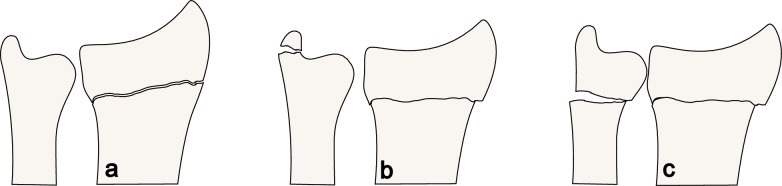
Example of distal radius and ulnar fractures as defined in this study. **a** Distal radius fracture only S52.5. **b** Distal radius fracture with avulsion of the ulnar styloid process (S52.5) **c** Distal radius and distal metaphyseal ulna fractures (S52.6)

There are two ICD-10 SE codes for distal forearm fracture: S52.5, isolated DRF (i.e. Colles fracture, Smith fracture) and S52.6, fracture of the distal end of both radius and ulna [12]. In more than half of all DRF cases there is an associated fracture of the ulnar styloid process [20], which represents an avulsion though ligaments (Fig. 1b) described as a concomitant injury in a Colles fracture [21]. In the ICD-10SE the definition of S52.5 and S52.6 respectively are not clearly defined. Many orthopaedic surgeons (including us) define a metaphyseal ulnar fracture as fracture on the distal end of ulna (S52.6), and an avulsion of the styloid process as part of a Colles’ fracture (S52.5) (Fig. 1). Both codes S52.5 and S52.6 were considered to correspond to a DRF for the purpose of the main analysis but were also investigated separately as an additional analysis.

### Data collection

Datasets of all health care visits reported with a DRF with or without an associated distal ulna fracture (ICD-10 S52.5, distal radius fracture or S52.6, distal radius and ulnar fracture) as a main or contributory diagnosis during 2001–2016 were provided by the Swedish National Board of Health and Welfare. A diagnosis of fracture was defined as the first time a patient appeared in the register within the study period. Visits appearing with an ICD-10 coding of S52.5 or S52.6 in the register after the initial visit were excluded in order to eliminate having a higher probability of being selected due to many healthcare visits. Bilateral fractures were counted as one fracture. Cohorts were constructed as presented in Table 1. The SPSS random generator was used to generate 240 simple random samples in each cohort. Each hospital was contacted by phone and formal requests of radiographic reports of the dates in question were sent by mail or telefax. All personal information was removed and replaced by a serial number.

### Validation of diagnosis

Diagnosis was validated by radiology reports retrieved from the treating hospital. In the cases where radiology reports failed to unequivocally confirm the diagnosis, medical records and/or radiographs were collected and reviewed. The diagnosis and the surgical code were considered valid when a radiology report, radiograph or medical records confirmed the diagnosis reported in the SNPR. We categorized the type of error in the same way as an investigation of the quality of the SNPR performed by the National Board of Health and Welfare [10]. Types of errors were: (a) Transfer to register error: ICD code in the SNPR does not correspond to the reported code in the medical record (b) Coding error: ICD code in the medical record does not correspond to the diagnosis written in plain text or (c) Diagnostic error: The diagnosis written in plain text does not correspond to the available medical information in the medical record.

### Statistics

A power analysis was performed to estimate the minimum sample size needed. Based on an assumed PPV of 90% and with the aim of a precision of ± 5 percent units within a 95% confidence interval 200 cases were needed to be included in each cohort. To account for an estimated 15% failure to retrieve radiographic reports or medical records the sample size was set to 240 cases per cohort.

Positive predictive value (PPV) was calculated as the number of patients with a confirmed diagnosis divided by the total number of participants. Confidence intervals of PPV were calculated with Clopper-Pearson. Clustered missing data from an entire hospital was considered missing at random (MAR) and units were excluded from the analysis. Single scattered missing data could be not missing at random (NMAR). Therefore, as a sensitivity analysis, scattered missing data was classified as no fracture or no surgery.

## Results

**Fig. 2:**
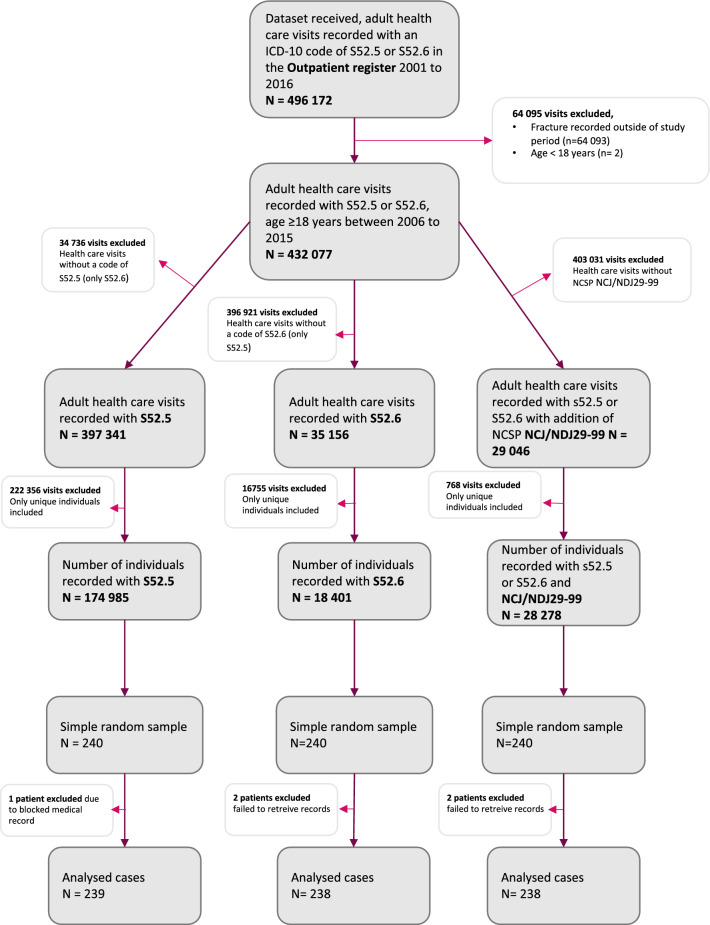
Flow chart describing selection of Cohort 1–3 in the Outpatient Register used in a validation study of the Swedish National Patient Register with regards to ICD-10 code registrations of distal radius fractures. S56.5 and S52.6 are not mutually exclusive which explains why 420 cases are included in both groups. NCSP classification of surgical procedures

**Fig. 3:**
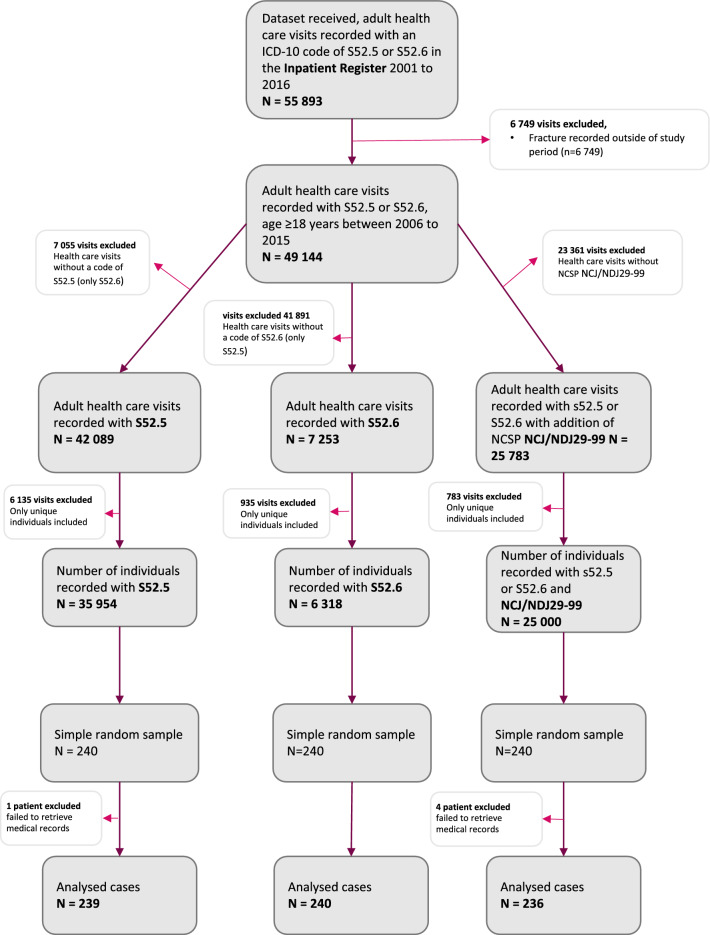
Flow chart describing selection of Cohort 4–6 in the Inpatient Register used in a validation study of the Swedish National Patient Register with regards to ICD-10 code registrations of distal radius fractures. S56.5 and S52.6 are not mutually exclusive which explains why 198 cases are included in both groups. NCSP -classification of surgical procedures

Between 2006 and 2015, 496 172 health care visits were reported to the Outpatient Register with an ICD-10 code of DRF, S52.5 or S52.6. The corresponding number for the Inpatient Register was 55 893. The cohorts and samples were constructed as presented in Figs. 2, 3. Of the 1440 sampled cases, medical and or radiographic reports were obtained in 1430 cases (99%) from 69 different sites. One hospital failed to contribute any data, giving a missing cluster of 8 units in total (1 in Cohort 2, 2 in Cohort 3, 1 in Cohort 4, 4 in Cohort 6). The other two missing cases consist of one patient whose medical record was inconclusive and additional information was not made available and one patient who refused to allow use of their medical records. The highest frequency of missing data was in Cohort 6 (all MAR, cluster missing from one hospital) reaching 1.7%. Basic characteristics of the study population as well as in all eligible cases are presented in Additional file 1: Appendix.

### Confirmation of diagnosis

Radiology and/or medical reports confirmed the diagnosis in a total of 1378 cases distributed unevenly between cohorts (Table 2). PPV of having any fracture involving distal radius ranged from 92% (S52.6 distal radius and ulna fracture) to 100% (S52.5/S52.6 and code for fracture surgery). As a sensitivity analysis the scattered missing cases were counted as no fracture in the two affected cohorts, but the PPV was not significantly affected (96.7% instead of 97.1% in Cohort 1 and 91.6% instead of 92.0% in Cohort 2). The PPV in Cohort 2 (S52.6 in the Outpatient Register) for an actual combined distal radius and distal metaphyseal ulnar fracture in was 35% and in Cohort 5 (S52.6 in the Inpatient Register) was 63%.

**Table 2:** Confirmed fractures, positive predictive value (PPV) and 95% confidence interval (CI) in a validation study of the Swedish National Patient Register with regards to ICD-10 code registrations of distal radius fractures

	Confirmed distal radius fracture (with or without distal ulna fracture)	S52.5 Distal radius fracture only	S52.6 Fracture of distal radius and distal ulna (defined as metaphyseal fracture)	Missing	No distal radius fracture
	PPV (95% CI)	PPV (95% CI)	PPV (95% CI)	Number	Number
Outpatient register					
Cohort 1, S52.5	97% (94–99)	94% (90–96)	3% (1.5–6.5)	1	8
Cohort 2, S52.6	92% (88–95)	57% (51–64)	35% (29–41)	2	19
Cohort 3, surgery	100% (99–100)	95% (91–97)	5% (3–9)	2	0
Inpatient register					
Cohort 4, S52.5	98% (95–99)	93% (88–96)	5% (3–9)	1	5
Cohort 5, S52.6	95% (92–98)	32% (26–38)	63% (57–69)	0	11
Cohort 6, surgery	96% (93–98)	79% (74–84)	17% (12–22)	4	9

A description of inaccurately coded cases is presented in Table 3. Out of the 52 unconfirmed cases 38 cases had another upper limb trauma, 1 had a foot trauma and 8 patients had complications of healed DRF (> 1 year since injury). The 47 cases of confirmed but incorrectly categorized trauma were categorized as coding error since one or both codes S52.5/S52.6 were present. One patient was initially suspected to have a nondisplaced DRF, but the suspicion was dismissed at10 day follow-up; this case was categorized as a diagnostic error. In 4 cases no code of S52.5 or S52.6 were found in the medical records and those cases were categorized as transfer to register error.

**Table 3:** Description of cases where distal radius fracture (DRF) was not confirmed in a validation study of the Swedish National Patient Register with regards to ICD-10 code registrations of distal radius fractures (DRF)

		Type of error		
	Total number of unconfirmed	Transfer to register error	Coding error	Diagnostic error
Cohort 1	8	No medical record corresponding with the site and date = 1	Hand injury = 1 Proximal forearm fracture = 1 Proximal humerus fracture = 1 Complication of a DRF of older date = 3	Wrist contusion = 1
Cohort 2	19	Knee injury, no diagnosis or code for DRF = 1	Hand injury = 3 Isolated distal ulna fracture = 9 Forearm shaft fracture = 2 Proximal forearm fracture = 1 Foot injury = 1 Complication of a DRF of older date = 2	
Cohort 3	0			
Cohort 4	5	Monteggia fracture – no coding for DRF = 1	Isolated distal ulna fracture = 1 Proximal forearm fracture = 1 Complication of a DRF of older date = 2	
Cohort 5	11	No code for DRF corresponding with the site and date = 1	Hand injury = 2 Isolated distal ulna fracture = 1 Forearm shaft fracture = 6 Complication of a DRF of older date = 1	
Cohort 6	9		Forearm shaft fracture = 3 Proximal forearm fracture = 5 Isolated distal ulna fracture = 1	

### Confirmation of surgical intervention

Of the 480 sampled cases with the additional NCSP code for fracture surgery, information of surgical intervention was retrieved in 474 cases (4 missing from the missing cluster). PPV for fracture surgery of DRF was 99.6% in the Outpatient Register and 95% in the Inpatient Register (Table 4).

**Table 4:** Validation of surgical codes for distal radius fractures (NCSP) in a validation study of the Swedish National Patient Register with regards registrations of distal radius fracture treatment

	Outpatient Register Cohort 3	Inpatient Register Cohort 6
Missing (%)	2 (0.8%)	4 (1.7%)
	Both from the missing cluster	All from the missing cluster
Confirmed surgery of the distal radius	238	225
No surgery	0	11
Surgical treated DRF PPV (95% CI)	100% (99–100)	95% (92–98)
Confirmed method of fracture surgery	235	224
Method of fracture surgery PPV (95% CI)	99% (96–100)	95% (91–97)

In the Outpatient Register all cases had had fracture surgery of a DRF, but three patients were reported with incorrect code for surgical method. One external fixation (NCJ/NDJ29) was coded as bioimplant (NCJ/NDJ39), one fixation with pins only was coded as combined methods and one plate and screws (NCJ/NDJ69) was coded as combined methods (NCJ/NDJ89).

There were eleven cases in the Inpatient Register who were registered as fracture surgery in the hand or forearm but had no surgical treatment of a DRF. Ten patients were treated surgically for proximal or diaphyseal forearm fractures, and one had surgery for a cervical femur fracture. Two of these patients had both a non-operatively treated DRF, and another fracture, operatively treated. Lastly one case was surgically treated for DRF but coded incorrectly. The registered surgical code was plate and screws, but the procedure actually performed was external fixation.

## Discussion

This study provides an excellent validity of the registrations of ICD-10 codes for DRF in the SNPR including coding for surgical interventions. PPVs for a correct diagnosis confirmed by radiology and/or medical reports were as high as 92–98%. Our findings support the general view of the SNPR as a reliable source for research data.

As for the code S52.6 (fractures to the distal radius and ulna, Fig. 1) the low PPV for distal metaphyseal ulna fracture indicates that fracture classification in the ICD-10 SE is not adequately defined. According to our results, the S52.6 in the SNPR cannot be used to analyse the occurrence of ulna fractures. On the other hand, PPV for a distal radius fracture with or without a concomitant distal ulnar fracture S52.5 and S52.6 was excellent. These findings show that classification systems rarely benefit from large number of subgroups, and that simplicity and clear descriptions of every class is necessary for correct use.

Waldenlind et al. [17] investigated the validity of incident rheumatoid arthritis by validating the first visit reported with the ICD code through chart reviews. In their study, 83% of registered cases were correctly coded while the remaining patients had other rheumatic conditions according to their medical records. Murley et. al [19] investigated the validity of multiple sclerosis in the In- and Outpatient Registers between 2001 and 2013 and confirmed 92,5% of the registered cases of multiple sclerosis in the SNPR. The authors suggested that the remaining patients with an uncertain diagnosis could possibly have represented cases where the diagnosis was initially suspected but later dismissed. This is supported by the fact that there are no single symptom or diagnostic tests that, alone, can diagnose these conditions. For a distal radius fracture, the debut is sudden with distinct clinical symptoms following a trauma. In addition, x-ray of the forearm in two planes is the gold standard for fracture detection and further clinical laboratory or radiological evaluation is rarely needed, thus we find it reasonable that the ICD-10 code for DRF has a high PPV.

Different fracture diagnoses have been validated in SNPR and been shown to have variable degrees of validity. Tampe et al. [22] found a PPV of 87% for open tibial fractures and Bergdahl et al. [23] validated acute humerus fractures to a PPV of 70%. The inclusion criteria could explain some of the differences. We only included first time occurrence since the code would appear repeatedly at the check-up. This must be taken under consideration when using data from the SNPR.

Our results differed considerably from the results presented in a study by Swärd et. al [16] investigating the validity of scaphoid fracture coding in the SNPR where a low PPV of 59% was presented. However, it is reasonable that the PPV is higher in our study and the discrepancy is believed to be caused by the difficulty of detecting a minimally or non-displaced scaphoid fracture on plain radiographs [24], making additional imaging necessary to correctly diagnose the patient. Usually, the additional imaging is performed in a subacute setting [16], making the first health care visit a subject of diagnostic and coding error.

### Strengths and limitations

The major strength of our study is its national coverage and the completeness of the data collection. We were able to retrieve 99% of the requested data for examination.

As a limitation our validation primarily relied on written radiology reports, and we did not review all x-ray images ourselves. However, radiology reports usually include the examination of x-ray images by two radiologists, and we find no reason to doubt their reports. Additionally we retrieved and reviewed the medical reports and/or x-ray images in cases where there was doubt regarding diagnosis.

Another limitation is that further analysis beside PPV is not possible due to the study design where the cases were identified through the register analysed. Thus, no true or false negative in the register could be found and neither negative predictive value, sensitivity nor specificity could be assessed.

## Conclusions

In conclusion, the validity of the diagnosis of distal radius fracture with or without an associated distal ulna fracture as well as the code for surgical intervention is high in the Swedish national outpatient and inpatients registers. According to our results, the register may be used as a reliable data source of population-based research of distal radius fractures.

### Supplementary Information

**Additional file 1: ** Demographics of patients in the Swedish National Patient Register with a distal radius fracture 2006–2015.

## Data Availability

According to the ethical approval and the sensitive personal data the data will not be readily available online but could deidentified data could be retrieved upon reasonable request.
