# Supplementary material for: Validity of distal radius fracture diagnoses in the Swedish National Patient Register
Source: Eur J Med Res. 2023 Sep 9;28:335. doi: 10.1186/s40001-023-01314-0 (PMC10492293; doi:10.1186/s40001-023-01314-0)
Supplement: Supplementary file 1 — Additional file 1: Demographics of patients in the Swedish National Patient Register with a distal radius fracture 2006–2015. [file 40001_2023_1314_MOESM1_ESM.docx]

| Table: Demographics of patients in the Swedish National Patient Register with a distal radius fracture 2006-2015. All cases refers the crudo demographics before randomly selecting the samples for the cohorts. | | | | | | | | |
| --- | --- | --- | --- | --- | --- | --- | --- | --- |
|  | Age | | | | | Gender | | |
|  | Mean age  all cases (CI) | Median age  all cases  (IQR) | Mean age sampled cases (95% CI) | Median age sampled cases  (IQR) | P-value  Mann Whitney U-set | Percentage females  All cases | Percentage females sampled cases | P-value  Chi-square test |
| Cohort 1 | 61.0 (60.9–61.1) | 63 (25) | 59.7 (57.4–62.1) | 62 (25) | 0.291 | 75.0% | 71.7% | 0,264 |
| Cohort 2 | 64.2 (64.0 – 64.5) | 66 (24) | 63.5 (61.1–66.0) | 66 (27) | 0.788 | 77.7% | 80.8% | 0.274 |
| Cohort 3 | 59.5 (59.3–59,6) | 62(17) | 61.7 (60.0–63.4) | 65 (14) | 0.007 | 80.1% | 85.8% | 0.028 |
| Cohort 4 | 667.1 (66.9-67.3) | 70 (25) | 66.5 (64.2-68.8) | 69 (26) | 0.506 | 76.1% | 73.8% | 0.403 |
| Cohort 5 | 69.5 (69.0-69.9) | 73 (23) | 67.3 (65.1-69.6) | 69 (25) | 0.029 | 80.5% | 77.5% | 0.259 |
| Cohort 6 | 62.8 (62.6-63.0) | 65 (21) | 62.8 (60.8-64.9) | 64(20) | 0.727 | 76.6% | 79.6% | 0.285 |
